# Development and application of a learning enjoyment scale for pedagogical activities

**DOI:** 10.12688/f1000research.147393.2

**Published:** 2024-07-31

**Authors:** Tarig Merghani, Rasha Babiker, Azza O. Alawad

**Affiliations:** 1 Physiology, RAK Medical and Health Sciences University, Ras Al-Khaimah, 11172, United Arab Emirates; 2 Physiology, Al-Neelain University, Khartoum, Sudan

**Keywords:** Pedagogical Activities, Learning Enjoyment Scale, Teaching Methods, Didactic Lectures, Teacher Proficiency

## Abstract

The impact of learning enjoyment on motivation, enthusiasm, and overall learning experiences is significant. Previous studies, lacking an unbiased tool for measuring enjoyment and confronting various influencing factors, produced conflicting results regarding enjoyment levels in different instructional methods. Hence, we developed a learning enjoyment scale for evaluating both active and passive educational activities. We applied the developed scale to 112 first-year medical and dental students to assess their enjoyment during didactic physiology lectures and explored possible associated factors. Within this data note, we present students’ responses to the developed LES. The LES encompasses six dimensions: knowledge, comprehension, application, analysis, concentration, and enjoyment. Students provided ratings for each dimension on a five-point Likert scale, spanning from 1 (strongly disagree) to 5 (strongly agree). The cumulative scores across the six dimensions range from a minimum of 6 to a maximum of 30. These total scores can be categorized as excellent (> 24), acceptable (18-24), or low (< 18). The second section of the dataset examines specific factors influencing overall enjoyment, such as teacher proficiency, topic difficulty, active student participation, objectives fulfillment, low stress levels, and self-perceived acquisition of skills. In addition to objective measurement of students’ enjoyment level, the LES can be utilized for quantitative cross-comparisons between different teaching activities. By employing this dataset, we will undertake an analysis to determine the internal consistency of the Learning Enjoyment Scale (LES), with the anticipation that the outcomes will be published in another venue.

## Introduction

The ongoing discourse on the effectiveness of teacher-centred learning approaches (passive instruction) versus student-centred learning methods (active instruction) persists, despite numerous studies conducted over the past three decades. Traditional lectures have been consistently criticized for their perceived lack of student engagement, largely due to their reliance on passive one-way information delivery. Students often view didactic lectures as dull or ineffective ^1^^–^^3^ when compared to the active learning approaches. Consequently, many educators have embraced strategies such as incorporating real-life scenarios, ^4^ introducing games, ^5^ utilizing technology, ^6^ or integrating problem-solving elements ^7^ to enhance the enjoyability of their lectures. However, the challenge persists, as there are no universally clear guidelines on how to structure lectures that are both educational and enjoyable.

Student Enjoyment is a state of psychological happiness experienced during learning. It is nicely defined by Hartley as the pleasant emotional state of the learner that develops during an educational activity due to experiencing a positive situation that motivates the learner to complete the task to persevere this feeling. ^8^ Although it is different from engagement, satisfaction or motivation, the combination contributes to a positive and effective learning atmosphere, fostering academic success and passion for knowledge. ^9^^,^^10^

Students’ enjoyment may be influenced by various factors, including but not limited to the instructor’s skill, the nature of the topic, and the educational environment. Furthermore, the diverse personalities and learning styles of students all contribute to and impact the level of enjoyment experienced during a teaching activity. ^11^^–^^13^

Due to the intricate nature of these variables and the significance of enjoyment in the learning process, there is a pressing need for an objective measurement tool to gauge students’ enjoyment across various teaching activities. Such a tool would not only streamline the assessment of diverse teaching methods but also aid in pinpointing the factors contributing to fluctuations in enjoyment levels within the same teaching method when presented by different instructors or for different subjects by the same faculty. Many educators tried different methods for measuring level of enjoyment during learning activities, ranging from a single question rating (for example from 0 to 6), ^14^ to different types of questionnaires such as satisfaction questionnaire with open ended questions, ^15^^,^^16^ adapted items from Achievement Emotions Questionnaire ^17^ and researcher-prepared questionnaire based on enjoyment indicators. ^18^ In this study, our primary objective is to develop a comprehensive learning enjoyment scale (LES) for the evaluation of both active and passive teaching activities. We applied the developed scale to 112 first-year medical and dental students, evaluating their enjoyment during didactic Physiology lectures and investigating potential associated factors. This data note presents the students’ responses to the developed LES. Our newly created scale, coupled with the associated dataset, ^1^ provides valuable insights into the nuances of student enjoyment, offering a tool that transcends the conventional dichotomy of teaching methods and delves into the factors shaping students’ learning experiences.

## Methods

**Figure 1.**
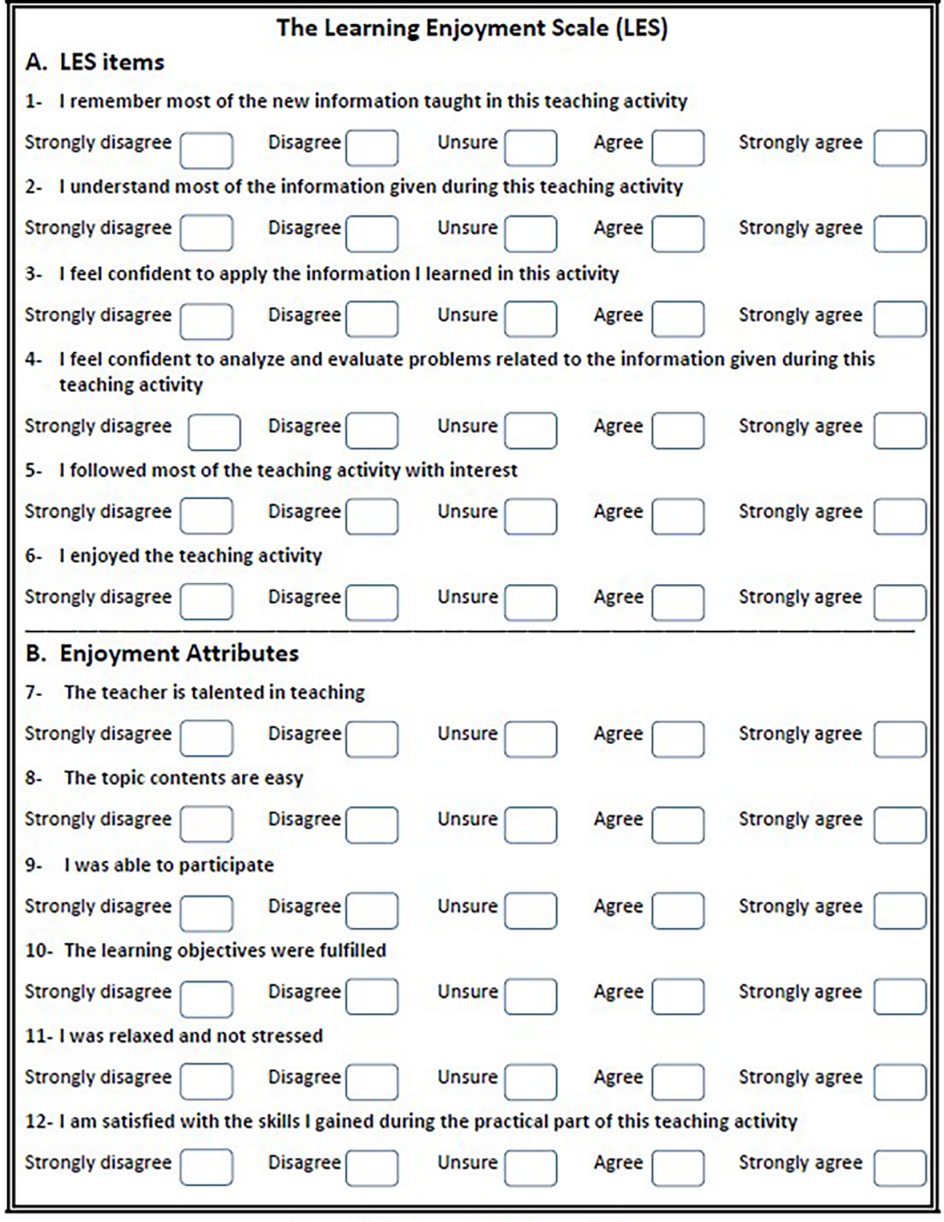
The learning enjoyment scale.

We developed the Learning Enjoyment Scale (LES) as a comprehensive and objective tool for measuring students’ enjoyment in the learning process, Figure 1. ^1^ The scale items are strategically aligned with the major categories of Bloom’s Taxonomy, emphasizing cognitive knowledge and affective attitude. Furthermore, the third domain, psychomotor skills, is partially assessed in the second section of the scale, highlighting its role as part of the overall enjoyment attributes. The LES first section consists of six items: knowledge, comprehension, application, analysis, concentration, and enjoyment. Students are instructed to rate each item on a five-point Likert scale, ranging from 1 to 5 (1 = strongly disagree, 2 = disagree, 3 = unsure, 4 = agree, and 5 = strongly agree). The minimum and total scores across the six items amount to 6 and 30, respectively. An excellent score is defined as above 80% (25-30), an acceptable score falls within the range of 60-80% (18-24), and a low score is considered less than 60% (<18). The 5-Likert scale selection and categorization into excellent, acceptable, and low is justified by its ability to provide clear interpretations of students’ enjoyment levels. In addition, the thresholds (60% and 80%) align with educational standards commonly used to assess performance and satisfaction levels in various academic contexts.

The second section of the LES evaluates the impact of various factors on students’ enjoyment. These factors encompass the teacher’s talent or proficiency, the complexity of the topic, student participation, objectives fulfillment, perceived stress levels, and the development of skills. Through direct analysis, the contribution of each factor to the overall LES is determined, offering a detailed understanding of the specific elements influencing students’ enjoyment during the learning process. The scoring system of the LES questionnaire aims to provide a quantitative assessment of the multifaceted aspects contributing to students’ enjoyment in educational settings.

The provided dataset represents students’ responses to the items of the developed scale. The data was collected from first-year undergraduate medical and dental students during two physiology lectures. We selected first-year students as they are newcomers to university education, suggesting a lack of established preferences for specific instructional methods at this early stage. A total of 112 university students, age ≥ 18 y contributed, with an overall participation rate of 68%. Prior to completing the questionnaire, students provided written informed consent to participate. The University Research Ethics Committee (RAKMHSU-HEC) granted approval for the study on 15 ^th^ November 2023, under the reference number “HEC-10-2023/24-F-M.”

Several limitations need to be taken into consideration when interpreting the results of this study. Firstly, the exclusive inclusion of first-year students raises the possibility that their perceptions may differ from those of students in later academic years. Additionally, with a response rate of 68%, the potential impact of non-participating students’ perspectives on the results remains uncertain. Furthermore, the preference for didactic lectures may be influenced by the different learning styles of the students, which are not shown in this dataset. ^13^ Lastly, additional investigation is warranted by applying this scale to different subjects, employing varied teaching methods, and exploring diverse geographic locations to enhance the generalizability of the scale. In conclusion, we believe that our scale contributes significantly to the medical education field by addressing the gap in measuring student enjoyment in educational contexts. Our full manuscript that contains enhanced findings, analysis and discussion will be published in another venue.

### Ethics and consent

The University Research Ethics Committee (RAKMHSU-HEC) granted approval for the study on 15 ^th^ November 2023, under the reference number “HEC-10-2023/24-F-M.”

Students provided written informed consent to participate.

## Data Availability

Zenodo: Development and Application of a Learning Enjoyment Scale for Pedagogical Activities, https://doi.org/10.5281/zenodo.10526239. ^19^ This project contains the following underlying data:
- Students’ responses to the developed LES: To assess students’ enjoyment during didactic lectures and explore possible associated factors. Zenodo: Development and Application of a Learning Enjoyment Scale for Pedagogical Activities, https://doi.org/10.5281/zenodo.10526239. ^19^ This project contains the following extended data:
- Learning Enjoyment Scale (LES): Developed for evaluating both active and passive educational activities. Data are available under the terms of the Creative Commons Zero (CC0 1.0 Public domain dedication).
