# Supplementary material for: Development and application of a learning enjoyment scale for pedagogical activities
Source: F1000Res. 2024 Jul 31;13:273. Originally published 2024 Apr 15. [Version 2] doi: 10.12688/f1000research.147393.2 (PMC11384189; doi:10.12688/f1000research.147393.2)
Supplement: Peer review history

# Reviewer report: Reviewer response for version 2

**DOI:** 10.5256/f1000research.169714.r309317

**Published:** 2024-09-09

**Authors:** Mohi Eldin Magzoub

**Affiliations:** 1 United Arab Emirates University, Al Ain, United Arab Emirates

The responses are appropriate and satisfactory

Are sufficient details of methods and materials provided to allow replication by others?

Yes

Is the rationale for creating the dataset(s) clearly described?

Yes

Are the datasets clearly presented in a useable and accessible format?

Yes

Are the protocols appropriate and is the work technically sound?

Yes

Reviewer Expertise:

This article aims at developing a useful tool for measuring student engagement and satisfaction

I confirm that I have read this submission and believe that I have an appropriate level of expertise to confirm that it is of an acceptable scientific standard.

# Reviewer report: Reviewer response for version 1

**DOI:** 10.5256/f1000research.161586.r300476

**Published:** 2024-07-22

**Authors:** Mohi Eldin Magzoub

**Affiliations:** 1 United Arab Emirates University, Al Ain, United Arab Emirates

This study introduced a new instrument called the Learning Enjoyment Scale, which is used to assess the degree of enjoyment experienced by students. It is derived from Bloom's taxonomy. The scale encompasses the majority of the taxonomy categories on the knowledge domain and few items on the affective domain.

 Nevertheless, the scale primarily serves to categorize learning objectives in order to facilitate course design and direct assessment. Thus, it aims to enhance the overall efficacy of the learning and teaching process, and not directly put an emphasis on enjoyment.

 Although enjoyment is not explicitly stated as an objective in the affective domain, it can be seen as a constituent of favorable attitudes and values towards learning. The affective domain encompasses various categories, such as perceiving events, reacting to phenomena, assigning value, organizing, and characterizing based on a set of values. Enjoyment can be understood as the process of appreciating and reacting to various experiences, where the active involvement and curiosity of students in educational tasks play a significant role.

 There are only four items in the scale that directly focus on enjoyment and satisfaction, which are questions 5, 6, 11, and 12.

 Other learning theories might be explored that nest suit student enjoyment such as experiential l learning self-determination, and constructivism theories. 

 Another issue with the methodology is the lack of clear objectives. It is unclear whether the goal is to validate the scale by assessing reliability and validity, or to present the performance of students based on the items of the scale. The objectives must be explicitly stated and appropriately included into this section. 

 Another concern is how the cut-off points for determining excellence, poor, and low are established.

 The utilization of the five-point Likert scale is not suitable in this context. The scale must adhere to a linear format instead than a categorized format. For example, a value of 3 for "unsure" indicates that it cannot be decided whether it is high or low. An optimal approach would be to employ a rating scale consisting of either 5 or 10 points, including a spectrum from poor to excellent. Subsequently, a composite score can be calculated based on this scale.

Are sufficient details of methods and materials provided to allow replication by others?

Yes

Is the rationale for creating the dataset(s) clearly described?

Yes

Are the datasets clearly presented in a useable and accessible format?

Yes

Are the protocols appropriate and is the work technically sound?

Yes

Reviewer Expertise:

This article aims at developing a useful tool for measuring student engagement and satisfaction

I confirm that I have read this submission and believe that I have an appropriate level of expertise to confirm that it is of an acceptable scientific standard, however I have significant reservations, as outlined above.

# Response

**Published:** 2024-07-26

**Authors:** Tarig Merghani

**Affiliations:** Physiology, RAKMHSU, Ras Al-Khaimah, Ras Al-Khaimah, United Arab Emirates

Thank you very much for the comprehensive review and valuable comments. We have carefully considered the points you raised and prepared an updated version of our data note (version 2). Please, find our detailed responses to the raised points below.

 Student Enjoyment is a state of psychological happiness experienced during learning. It is nicely defined by Hartley as the pleasant emotional state of the learner that develops during an educational activity due to experiencing a positive situation that motivates the learner to complete the task to persevere this feeling (1). It is different from engagement, satisfaction or motivation. Since it is subjective feeling, there is no widely accepted scale for assessing enjoyment. Authors tried different methods ranging from a single question rating (for example from 0 to 6) (2), to different types of questionnaires such as satisfaction questionnaire with open ended questions (3, 4), adapted items from Achievement Emotions Questionnaire (5) and researcher-prepared questionnaire based on enjoyment indicators (6). In our scale, we utilized direct questions to measure enjoyment, and we added other questions to assess the students’ perception of different domains of learning (knowledge, comprehension, application, and analysis) which are known to be influenced by enjoyment (Q1 to 6). Other questions (Q7 to 12) are not intended to measure the enjoyment, they are intended to study certain factors that are known to influence enjoyment. The primary objective of this study is to develop a comprehensive learning enjoyment scale for the evaluation of both active and passive teaching activities. We sought to apply this scale objectively to measure the enjoyment scores of Physiology lectures and investigate associated factors. As suggested by the reviewer, we clearly mentioned the objectives of our study in the updated version of the article.

 The suggestion to explore other learning theories like experiential learning, self-determination theory, and constructivism is well-taken. We plan to incorporate discussions on how these theories could complement our findings on student enjoyment and educational outcomes in future studies. We also have the idea to revise and update our scale in the future based on feedback from reviewers and valuable suggestions from colleagues.

 The Learning Enjoyment Scale (LES) employs a five-point Likert scale (1 = strongly disagree to 5 = strongly agree) across its six dimensions, yielding total scores ranging from 6 to 30. The 80% represents the fourth option of the Likert scale (agree = 4), as 4/5 x 100%= 80%. Therefore, scores above 80% generally indicate that all responses are agree or above. The 60% represents the third option (unsure= 3), as 3/5 x 100%= 60%. Therefore, scores below 60% generally indicate that all the responses are disagree or below. The 5-Likert scale selection and categorization into excellent, acceptable, and low is justified by its ability to provide clear interpretations of students' enjoyment levels. In addition, the thresholds (60% and 80%) align with educational standards commonly used to assess performance and satisfaction levels in various academic contexts.  

 In conclusion, we believe that our scale contributes significantly to the medical education field by addressing the gap in measuring student enjoyment in educational contexts. We appreciate the opportunity to revise our manuscript based on the reviewer's valuable feedback and look forward to sharing our full manuscript that contains enhanced findings, analysis and discussion with the academic community in the near future.

 Thank you once again for your thorough evaluation and insightful comments.

 Best regards,

 Tarig Merghani

**References:**

1. Hartley D. Excellence and enjoyment: The logic of a ‘contradiction’. *British Journal of Educational Studies* 2006; 54(1):3-14.  
https://doi.org/10.1111/j.1467-8527.2005.00331.x
2. Griffee DT. Connecting Theory to Practice: Evaluating a Brain-based Writing Curriculum. *Learning Assistance Review* 2007;12(1):17-27
3. Marín-Vinuesa LM, Rojas-García P. Expected Usefulness of Interactive Learning Platforms and Academic Sustainability Performance: The Moderator Role of Student Enjoyment. *Sustainability*. 2024; 16(9):3630. https://doi.org/10.3390/su16093630
4. Dehghan S, Horan EM, Frome G. Investigating the Impact of the Flipped Classroom on Student Learning and Enjoyment in an Organic Chemistry Course. *Journal of Chemical Education* 2022; 99(7):2512-2519. https://doi.org/10.1021/acs.jchemed.1c01104 
5. Bieleke M, Gogol K, Goetz T, Daniels L, Pekrun R. The AEQ-S: a short version of the achievement emotions questionnaire. Contemp. *Educ. Psychol.* 2021; 65:101940. https://doi.org/10.1016/j.cedpsych.2020.101940
6. Mirawati M, Sikarni W. Description of Student Attitudes: Enjoyment in Learning Physics and Interest in More Time Studying Physics. Sch. Jo. Phs. Ed [Internet]. 2023 Mar.31 [cited 2024Jul.24];4(1):1-. Available from: https://cahaya-ic.com/index.php/SJPE/article/view/490

# Reviewer report: Reviewer response for version 1

**DOI:** 10.5256/f1000research.161586.r268529

**Published:** 2024-05-17

**Authors:** Tarig Fadelelmoula

**Affiliations:** 1 National University of Science and Technology, Muscat, Oman

Existing student learning enjoyment evaluation scales are not sufficiently tested. The objective of this project is to develop and apply a new learning enjoyment scale for evaluating students’ enjoyment during different pedagogy sessions. The researchers designed the tool in accordance with Bloom's taxonomy. The tool had two sections: (1) A six-domain Likert scale, (2) examining specific factors influencing overall enjoyment, such as teacher proficiency, topic difficulty, active student participation, objectives fulfillment, low stress levels, and self-perceived acquisition of skills. The scale was implemented in year 1 medical and dental students. The initial analysis of data reflected the internal consistency of the scale; however, the results are limited by the low response rate, the confinement to didactic lectures, and student level. Further research is needed to include a larger group of students, check the reliability of the scale, and test it in student centered pedagogies.

Are sufficient details of methods and materials provided to allow replication by others?

Yes

Is the rationale for creating the dataset(s) clearly described?

Yes

Are the datasets clearly presented in a useable and accessible format?

Yes

Are the protocols appropriate and is the work technically sound?

Yes

Reviewer Expertise:

Pulmonology, Physiology, Medical Education, Healthcare Simulation

I confirm that I have read this submission and believe that I have an appropriate level of expertise to confirm that it is of an acceptable scientific standard.

## References

[1] : The effects of personalized gamification on students’ flow experience, motivation, and enjoyment. *Smart Learning Environments* .2022;9(1) : 10.1186/s40561-022-00194-x 10.1186/s40561-022-00194-x

[2] : THE EFFECT OF ENJOYMENT ON LEARNING.2018; 10.21125/inted.2018.1087 508-514 10.21125/inted.2018.1087

# Response

**Published:** 2024-06-10

**Authors:** Tarig Merghani

**Affiliations:** Physiology, RAKMHSU, Ras Al-Khaimah, Ras Al-Khaimah, United Arab Emirates

Many thanks for the review comments. We appreciate your insights and feedback on our data note. Further results and analysis will be presented in a full article (under review).

 We acknowledge that existing student learning enjoyment evaluation scales have not been sufficiently tested, which is why our project aimed to fill this gap by designing a comprehensive tool based on Bloom's taxonomy. We are pleased that the initial analysis reflected the internal consistency of the scale. However, we also recognize the limitations you pointed out, namely the low response rate, the confinement to didactic lectures, and the restriction to year 1 medical and dental students. We are planning to address these limitations by including a larger and more diverse group of students. Thank you once again for your valuable feedback.

 Regards

 Tarig Merghani
